# Supplementary material for: Efficacy of 1-Kestose Supplementation in Patients with Pancreatic Ductal Adenocarcinoma: A Randomized Controlled Pilot Study
Source: Nutrients. 2024 Aug 29;16(17):2889. doi: 10.3390/nu16172889 (PMC11397247; doi:10.3390/nu16172889)
Supplement: Supplementary file 1 [file nutrients-16-02889-s001.zip › nutrients-3156051-supplementary.pdf]

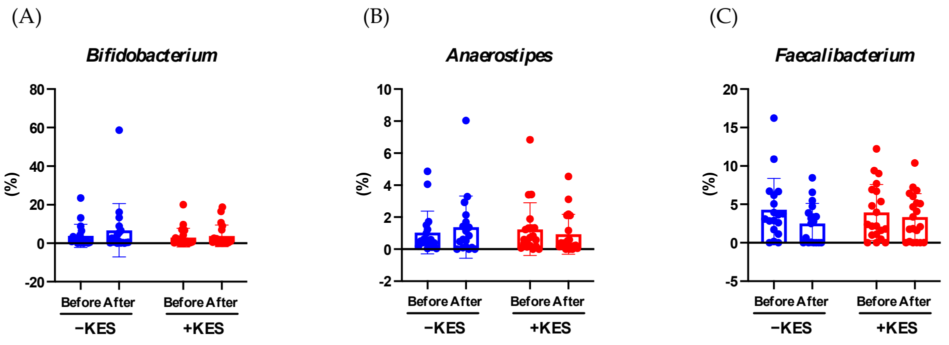

Figure S1. Analysis of the gut microbiota before and after treatment in the −KES and +KES groups. Data are presented as mean ± SD. (A) *Bifidobacterium*, (B) *Anaerostipes*, (C) *Faecalibacterium*. The Wilcoxon test was utilized to calculate statistical significance. −KES, group not administered 1-kestose; +KES, group administered 1-kestose.
